# The Impact of Glycemic Control on Ranibizumab Efficacy in Diabetic Retinopathy: A Retrospective Analysis

**DOI:** 10.7759/cureus.77124

**Published:** 2025-01-08

**Authors:** Chebly Dagher, Maria Akiki, Rita Saliby, Georges Harb

**Affiliations:** 1 Internal Medicine, University of Connecticut School of Medicine, Hartford, USA; 2 Internal Medicine, Univeristy of Connecticut, Farmington, USA; 3 Endocrinology, Holy Spirit University of Kaslik, Kaslik, LBN; 4 Ophthalmology, Faculty of Medicine, Lebanese University, Beirut, LBN

**Keywords:** best corrected visual acuity, central retinal thickness, glycosylated hemoglobin, macular edema, ranibizumab

## Abstract

Background: Diabetic retinopathy (DR), a leading cause of vision loss, is driven by inflammation, oxidative stress, and vascular endothelial growth factor (VEGF) production, with elevated blood glucose and advanced glycation end products (AGEs) exacerbating retinal damage. While intravitreal VEGF inhibitors have become the first-line treatment for diabetic macular edema (DME), response to therapy varies due to systemic factors such as HbA1c levels, blood pressure, and diabetes duration.

Objectives: This study aims to assess the impact of glycosylated hemoglobin (HbA1c) control on the effectiveness of Ranibizumab treatment in patients with DR.

Methodology: The study included 222 eyes from 222 patients with type 2 diabetes, comprising 60% males and 40% females, with an average age of 60.2 ± 9.32 years. Participants were administered monthly intravitreal injections of Ranibizumab, 0.05 mL of a 6 mg/mL solution, over three months, and an optical coherence tomography (OCT) was done one month after the treatment for the evaluation of the patient's need for further injections. HbA1c, central retinal thickness (CRT), and best-corrected visual acuity (BCVA) were measured at the beginning and end of the study. Patients were then divided into two groups according to their HbA1c level, with a cut-oﬀ point of 7% (53 mmol/mol).

Results: At the beginning of the study, the HbA1c mean was 8.16% ± 1.2%, the BCVA mean was 59.7 ± 9.73, and the central macular thickness (CMT) mean was 465.4 ± 132.34 µm. Twelve months later, the 222 patients were separated into two groups based on HbA1c levels: 109 patients had an HbA1c >7% (group A) and 113 patients had an HbA1c ≤7% (group B). The improvement of BCVA was 6.1 ± 7.3 for group A versus 7.9 ± 6.1 for group B (*P* = 0.0478). The reduction in CMT was 164.2 ± 122.8 μm for group A versus 197.8 ± 125.1 μm for group B (*P* = 0.0447).

Conclusions: Our study indicates that HbA1c control influences the treatment outcomes of intravitreal Ranibizumab for DME, with better responses observed in patients whose HbA1c is below 7%.

## Introduction

Diabetes mellitus (DM) is a driving cause of mortality and morbidity around the world [1]. A major problem of DM is retinopathy contributing to visual loss [2]. According to the National Eye Institute, diabetic retinopathy (DR) is the major reason for vision loss in adults in the United States [3]. The initial triggers of DR are manifested by inflammation and oxidative stress. Inflammation results in cellular migration, leucocyte adhesion, platelets aggregation, and capillary basement membrane thickening, impending retinal blood flow. As a result, tissue hypoxia increases the production of vascular endothelial growth factor (VEGF).

Furthermore, increased blood glucose levels contribute to oxidative stress by increasing the genesis of reactive oxygen species (ROS). Advanced glycation end products (AGEs) are one of the biochemical pathways that favor retinal oxidative stress [4].

The generation of AGEs is a part of normal metabolism. However, high levels of AGEs in tissues can become pathogenic. In the eyes, AGEs damage retinal microvasculature leading to the production of VEGF [5]. VEGF may moreover have a part within the etiology of diabetic macular edema (DME) due to its activity in enhancing capillary permeability [4].

The occurrence rises from 0% to 3% in patients newly diagnosed with DM to 29% in those with chronic DM (>20 years) [6]. The overexpression of VEGF in diabetic patients plays an important part in the progression of DME [7], thereby disfigurement of visual images that may lead to a remarkable reduction in visual acuity (VA).

VA measurement is based on the point-by-point, thorough refraction and VA conventions of the Early Treatment Diabetic Retinopathy Study (ETDRS), broadly considered the gold standard for evaluating VA within the ophthalmic clinical [7].

CMT is obtained through OCT, a computerized machine that produces images of the retinal structure and the vitreoretinal interface [8]. Historically, laser photocoagulation was the essential strategy utilized to treat vision complications [9]. In contrast, anti-VEGF treatment has been indicated to be safer than laser photocoagulation therapy [10]. Moreover, a noticeable improvement in visual and anatomic response was seen during the treatment of DME [11] with the use of intravitreal VEGF inhibitors. Consequently, this progress led to a change in the standardized management of DME, which resulted in intravitreal VEGF inhibitors becoming the first-line treatment for patients with DME [12,13]. Despite these favorable results, the advantages of the previously mentioned treatments turned out to be inconstant between patients. Early investigations have illustrated the significance of systemic variables for the progression of DR and the decrease in VA. Many local and systemic variables, such as duration of DM, HbA1c level, blood pressure control, microalbuminuria, and dyslipidemia, may contribute not only to the development of DME but also to the treatment outcome [14]. The higher the HbA1c level, the greater the risk of DME [15,16].

The aim of this study was to assess the efficacy of glycosylated hemoglobin control on the response of Ranibizumab in patients with DR. We assessed the effect of HbA1c levels on the reduction of macular edema and visual outcomes following Ranibizumab treatment in patients with DME.

## Materials and methods

A total of 222 patients with DME were included in a retrospective observational study after providing informed/written consent. The study was approved by the institutional review board of Hopital Francais du Levant. Pretreatment clinical variables included were best-corrected visual acuity (BCVA), dilated fundus examination, intraocular pressure (IOP), central macular thickness (CMT), and serum HbA1c.

The study included patients experiencing visual impairment due to DME. They were 18 years old or above, had type 2 diabetes mellitus with an HbA1c level of 10% or less, a BCVA score between 78 and 39 letters (Early Treatment Diabetic Retinopathy Study [ETDRS]), and a CME of more than 300 µm. Those excluded from the study were patients using concomitant medications that could prevent the improvement of VA, having an active intraocular inflammation, infection, or glaucoma in either eye. Patients were not included if they had a history of stroke or untreated or uncontrolled hypertension. Patients who underwent pan-retinal laser photocoagulation in the affected eye within 6 months before or during the study were excluded (Table 1).

**Table 1:** Inclusion and exclusion criteria. CMT, central macular thickness; BCVA, best-corrected visual acuity; ETDRS, Early Treatment Diabetic Retinopathy Study; Hba1c, hemoglobin A1c (glycated hemoglobin); DME, diabetic macular edema; VA, visual acuity

Inclusion criteria	Exclusion criteria
Adults ≥18 years of age	Active intraocular inflammation in either eye
Diabetes mellitus	Any active infection in either eye
CMT >300 µm	Uncontrolled glaucoma in either eye
BCVA score between 78 and 39 letters (ETDRS)	History of stroke
Hba1c level 10% or less	Untreated or uncontrolled hypertension
Visual impairment resulting from DME	Pan-retinal laser photocoagulation in the study eye within six months before or during the study
Decreased VA resulting from DME and not from any other disease	The use of concomitant medications that could prevent the improvement of VA during the study

Participants were administered monthly intravitreal injections of Ranibizumab, 0.05 mL of a 6 mg/mL solution, over three months and were followed up after 1 month with an OCT and a VA test. A decision on the need for further injections was made based on the results. If the decision was to wait, the same procedure was repeated every month until 12 months.

Baseline HbA1c analysis

To evaluate the efficacy of HbA1c control on the effect of Ranibizumab in patients with DR, participants were categorized into two groups based on HbA1c levels: those with HbA1c <7% (<53 mmol/mol) and those with HbA1c >7% (>53 mmol/mol).

Statistical analysis

Statistical analyses were conducted to evaluate data in terms of range, case numbers, mean ± SD, and percentages as appropriate. A paired t-test was employed to determine the mean differences in VA and CMT. Additionally, the study population was divided into two groups based on HbA1c values: ≤7% (<53 mmol/mol) and >7% (≥53 mmol/mol), to assess treatment effects in different HbA1c subgroups. The mean changes from baseline in BCVA and CMT were compared between these subgroups using an independent t-test. A *P*-value of <0.05 was deemed statistically significant. Statistical analyses were conducted using Microsoft Excel and SPSS (IBM Corp., Armonk, NY).

## Results

A total of 222 eyes from 222 patients with type 2 DM were included in the study, of which 60% were males and 40% were females, with a mean age of 60.2 ± 9.32 years. The mean duration of diabetes was 11.33 ± 8.85 years. The mean time since the first diagnosis of DME was 1.36 ± 2.08 years. The mean level of HbA1c baseline was 8.16% ± 1.2% and about 113 (50.9%) had HbA1c >7% (>53 mmol/mol), the remaining (109, 49.09%) had HbA1c <7% (<53 mmol/mol). The mean number of Ranibizumab injections was 7.8 ± 2.94, with 27 (12%) patients receiving three injections, 49 (22%) patients receiving six injections, and 146 (66%) receiving nine injections. At baseline, the mean BCVA was 59.7 ± 9.73 and improved to 6.1 ± 7.3 in group A and to 7.9 ± 6.1 in group B at 12 months, with *P* = 0.0478. The mean baseline CMT was 465.4 ± 132.34 µm and decreased to -164.2.13 ± 122.8 µm in group A and to -197.8 ± 125.1 µm in group B at 12 months, with *P* = 0.0447. The results are summarized in Table 2.

**Table 2:** Comparison of BCVA and CMT between groups A and B at baseline and 12 months. BCVA, best-corrected visual acuity; EDTRS, Early Treatment Diabetic Retinopathy Study; CMT, central macular thickness; SD, standard deviation

Measure	Baseline (Mean ± SD)	At 12 months, Group A (Mean ± SD)	At 12 months, Group B (Mean ± SD)	t-test statistic	*P*-value
BCVA (EDTRS)	59.7 ± 9.73	6.1 ± 7.3	7.9 ± 6.1	-1.99	0.0478
CMT (µm)	465.4 ± 132.34	164.2 ± 122.8	197.8 ± 125.1	-2.02	0.0447

**Figure 1:**
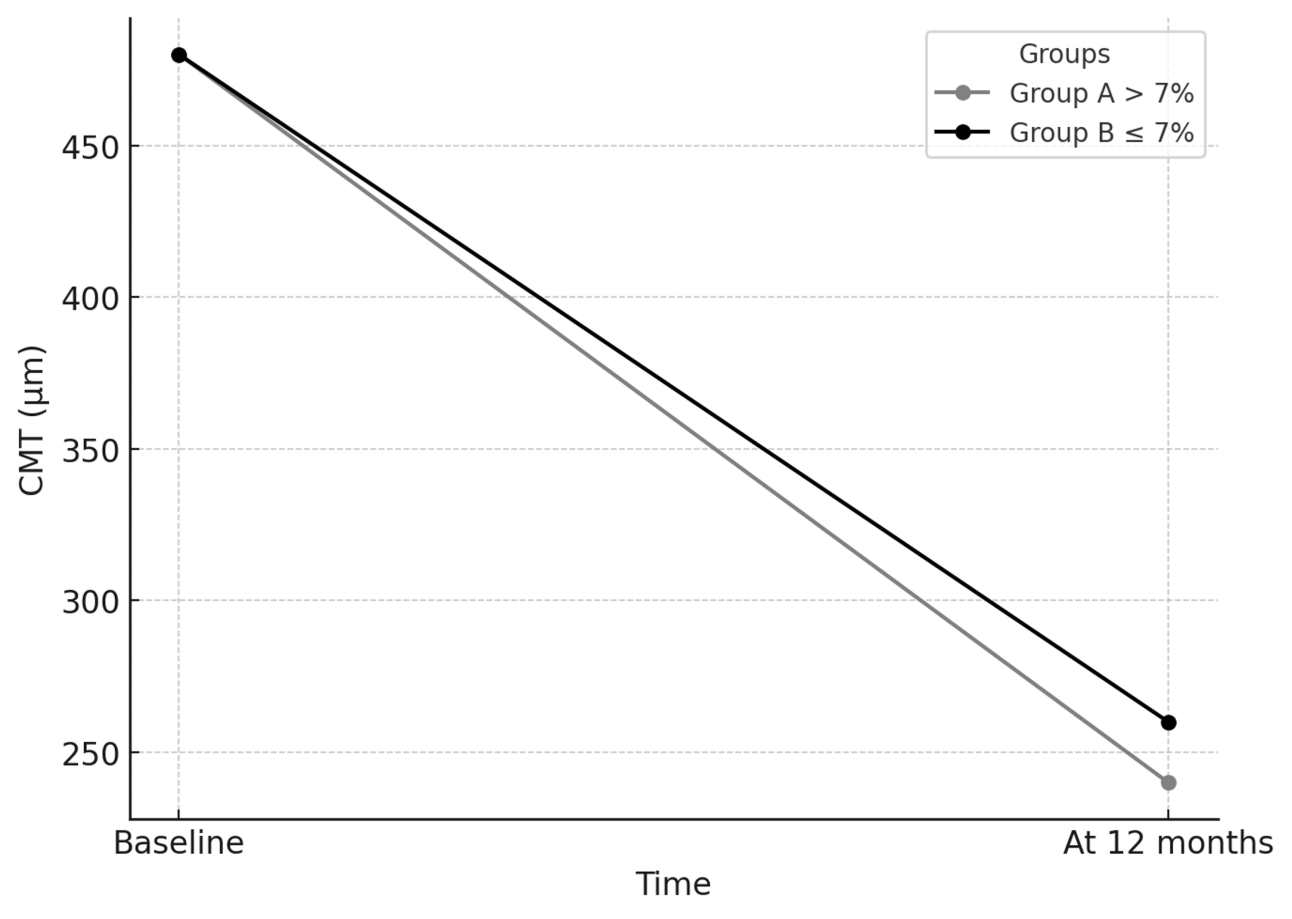
Central macular thickness (CMT) over time.

The average difference in CMT reduction between the two groups was 33.6 μm, which was statistically significant (*P *= 0.0447), as illustrated in Figure 1.

**Figure 2:**
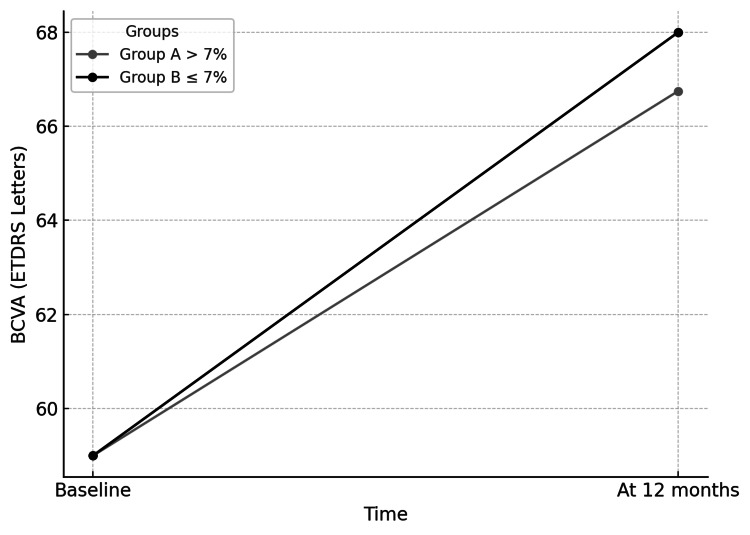
Best-corrected visual acuity (BCVA) over time. ETDRS, Early Treatment Diabetic Retinopathy Study (BCVA measured in ETDRS letters)

The average change in BCVA was 1.8, which was statistically significant (*P *= 0.478), as demonstrated in Figure 2.

## Discussion

Our study has shown that the control of HbA1c results in improvement of macular thickness and VA in diabetic patients with DR treated with Ranibizumab. We found that higher levels of HbA1c were associated with greater CMT and decreased BCVA compared to lower levels after 12 months of treatment with Ranibizumab. Moreover, multiple studies focus on the significance of glycemic control on the advancement of DME. Only a few studies have shown the role of glycemic control in influencing the response to VEGF inhibitor treatment.

Adults with vision loss from DME and a CMT ≥ 275 μm were included in phase 3 randomized trials, RISE and RIDE, to study the effect of Ranibizumab intravitreal injections while evaluating the percentage of patients acquiring ≥15 letters in BCVA from baseline at two years [17]. In RIDE, 33.6% of 382 patients receiving 0.3-mg Ranibizumab and 45.7% receiving 0.5-mg Ranibizumab showed ≥15 letters gained in BCVA from baseline at 2 years, compared to 12.3% of sham patients. Almost similar results were obtained in RISE. In the conclusion of both studies, Ranibizumab 0.3 mg and 0.5 mg significantly decreased vision loss and macular edema in patients with DR [17].

Sharma et al. [18] reported the effect of baseline HbA1c in patients with DR treated with Bevacizumab. Thirty-seven patients were included in this study having DME and were treated with monthly Bevacizumab injections. The study concluded that the baseline HbA1c control can influence the treatment outcome in further reducing the DME with a *P*-value < 0.00118. In our study, the anti-VEGF used was Ranibizumab, while evaluating the treatment outcome with a glycemic control 12 months after starting the injections.

In a prospective study involving 38 patients treated with Bevacizumab, Warid et al. [19] found that patients with HbA1c <7% (<53 mmol/mol) gained two lines of VA compared to those with HbA1c >7% (>53 mmol/mol), with a *P*-value <0.001. They concluded that poorer glycemic control may lead to worse visual outcomes.

Wong et al. [20] reported in a prospective observational study of 35 patients that systemic factors could affect the improvement of anti-VEGF therapy such as HbA1c, serum VEGF levels, lipidemia, renal clearance, and blood pressure. They concluded that lower HbA1c levels (7% or less) were highly correlated with a greater decrease in CMT at 30 days after injection of Bevacizumab or Ranibizumab [20]. In our study, we focused on only the importance of glycemic control and its effect on CMT and BCVA in treatment response to Ranibizumab injection while excluding patients with uncontrolled or untreated arterial blood pressure.

In a post hoc analysis by Shalchi et al. [21], there was no correlation between the increase in VA or the decrease in CMT and the change in HbA1c level or the initial level in patients treated with monthly Ranibizumab injection [21]. A total of 483 patients were divided into two groups based on their initial HbA1c level: <7% or >7%. Vision and CMT were significantly unchanged between the two groups at the 36-month follow-up [21].

In contrast to our results, a retrospective study of 119 patients by Rees et al. [22] stated that HbA1c is not a predictor of outcome in diabetic patients with DME receiving Ranibizumab injections. The improvement in BCVA and the mean number of treatment injections were similar in both the good and poor control groups. However, CMT was not included in their study [22].

In addition, the Moorfields OpenEyes database was used for a retrospective cohort study of 312 patients over one year at multiple hospitals to investigate the influence of HbA1c on Ranibizumab injections for DME [19]. As a result, HbA1c was not related to changes in VA or CMT, with P-values of 0.0577 and 0.099, respectively [19]. In conclusion, HbA1c was not related to functional or anatomical changes at one year in DR treated with Ranibizumab, in contrast to our study, which demonstrated a significant improvement in both VA and CMT.

The end-point depends on three variables: the follow-up duration, the choice of anti-VEGF injections, and its number. It is important to mention that the complexity of DME comes from the fact that its condition depends on multiple systemic and local factors, including primarily HbA1c and VEGF, cholesterol levels, and metabolic syndrome. Further studies are needed to demonstrate the efficacy of controlling each risk factor to achieve optimal outcomes in the treatment of DR. Although the overall mean metabolic parameters remained relatively stable during the study period, patients with uncontrolled or untreated arterial blood pressure were excluded.

The study has several limitations that should be considered. First, it was conducted at a single center, which may limit the generalizability of the findings to broader populations and diverse clinical settings. Second, the sample size of 222 patients, while sufficient for preliminary analyses, may restrict the statistical power to detect subtle differences in treatment outcomes. Additionally, the relatively short follow-up period of one year may not adequately capture the long-term effects of Ranibizumab treatment on VA and macular thickness. Furthermore, VA, although a commonly used measure, is inherently subjective and may introduce variability in the reported outcomes. These factors highlight the need for further studies with larger, multicenter cohorts and extended follow-up to validate and enhance the robustness of the findings.

## Conclusions

In conclusion, intravitreal Ranibizumab treatment has demonstrated significant improvements in VA and a reduction in macular thickness for patients with DME. A key factor influencing these outcomes is glycemic control, with the most favorable responses observed in patients maintaining an HbA1c level below 7%. Achieving strict blood glucose regulation not only optimizes the therapeutic effects but may also decrease the frequency of required injections, leading to better patient compliance. Thus, integrating glycemic management into treatment plans is essential for maximizing the benefits of Ranibizumab therapy.
